# Publisher Correction: Extracellular-matrix mediated stresses spatially bias myoblast fusion and myotube growth

**DOI:** 10.1038/s41467-026-79196-z

**Published:** 2026-10-29

**Authors:** Yoann Le Toquin, Sushil Dubey, Aleksandra Ardaševa, Lakshmi Balasubramaniam, Emilie Delaune, Valérie Morin, Amin Doostmohammadi, Christophe Marcelle, Benoît Ladoux

**Affiliations:** 1 Université Lyon 1, CNRS, Inserm, MeLiS, UMR 5284 – U1314, Lyon, France; 2 Department of Physics, Friedrich-Alexander Universität Erlangen-Nürnberg, Erlangen, Germany; 3 Max Planck Zentrum für Physik und Medizin and Max Planck Institute for the Science of Light, Erlangen, Germany; 4 Université Paris Cité, CNRS, Institut Jacques Monod, F-75013 Paris, France; 5 Niels Bohr Institute, University of Copenhagen, Copenhagen, Denmark; 6 Institute of Bioengineering, School of Life Sciences, École Polytechnique Federale de Lausanne (EPFL), Lausanne, Switzerland; 7 Wellcome Trust / CRUK Gurdon Institute, University of Cambridge, Cambridge, United Kingdom; 8 Monash University, Australian Regenerative Medicine Institute, Clayton, VIC Australia

**Keywords:** Biophysics, Mechanotransduction, Myosin

Correction to: *Nature Communications* https://doi.org/10.1038/s41467-026-76967-6, published online 22 August 2026

In the version of this article initially published, the red dots in Fig. 4m were misaligned, appearing on the fourth row of orientation lines rather than between the third and fourth rows. The figure has been updated in the HTML and PDF versions of the article. For comparison, the original figure is available as Supplementary Information accompanying this amendment.

## Supplementary information

Original Fig. 4

