# Supplementary material for: Extracellular-matrix mediated stresses spatially bias myoblast fusion and myotube growth
Source: Nat Commun. 2026 Aug 22;17:10057. doi: 10.1038/s41467-026-76967-6 (PMC13594122; doi:10.1038/s41467-026-76967-6)
Supplement: Supplementary file 2 [file 41467_2026_76967_MOESM13_ESM.pdf]

nature portfolio

Peer Review File

Extracellular matrix mediated stresses spatially bias myoblast fusion and myotube growth

Corresponding Author: Professor Benoit Ladoux

This manuscript has been previously reviewed at another journal. This document only contains information relating to versions considered at Nature Communications.

This file contains all reviewer reports in order by version, followed by all author rebuttals in order by version.

Version 0:

Reviewer comments:

Reviewer #1

(Remarks to the Author)
I believe the authors significantly improved their manuscript and I appreciated the futher analyses provided. I believe the data is more clearly presesented now, with stronger supporting evidence for the role of tissue mechanics and ECM nematics in spatially biasing myoblast fusion. The new analysis also provide a stronger framing to the authors interpretation and I appreciated the changes to the title and abstract.

Open Access This Peer Review File is licensed under a Creative Commons Attribution 4.0 International License, which permits use, sharing, adaptation, distribution and reproduction in any medium or format, as long as you give appropriate credit to the original author(s) and the source, provide a link to the Creative Commons license, and indicate if changes were made.
In cases where reviewers are anonymous, credit should be given to 'Anonymous Referee' and the source.
The images or other third party material in this Peer Review File are included in the article’s Creative Commons license,

unless indicated otherwise in a credit line to the material. If material is not included in the article's Creative Commons license and your intended use is not permitted by statutory regulation or exceeds the permitted use, you will need to obtain permission directly from the copyright holder.
To view a copy of this license, visit https://creativecommons.org/licenses/by/4.0/

REVIEWERS' COMMENTS

Reviewer #1 (Remarks to the Author):

I believe the authors significantly improved their manuscript and I appreciated the futher analyses provided. I believe the data is more clearly presented now, with stronger supporting evidence for the role of tissue mechanics and ECM nematics in spatially biasing myoblast fusion. The new analysis also provide a stronger framing to the authors interpretation and I appreciated the changes to the title and abstract.

We thank for the reviewer for their support.
