# Supplementary material for: Extracellular-matrix mediated stresses spatially bias myoblast fusion and myotube growth
Source: Nat Commun. 2026 Aug 22;17:10057. doi: 10.1038/s41467-026-76967-6 (PMC13594122; doi:10.1038/s41467-026-76967-6)
Supplement: Supplementary file 4 [file 41467_2026_76967_MOESM2_ESM.pdf]

Description of Additional Supplementary Files

Supplementary Movie 1: Chicken primary myoblasts transition to myotube in culture. Evolution of a primary chicken myoblasts culture along a 72h period. Scale bar, 500 µm.

Supplementary Movie 2: Time lapse of a motile +1/2 defect from birth to annihilation. Bright-field overlaid with averaged local orientation of the cells (red lines). +1/2 defects (magenta dots) and -1/2 defects (yellow triangle) merge together, leading to their annihilation. Scale bar, 100 µm.

Supplementary Movie 3: Effect of blebbistatin treatment on primary chicken myoblasts in culture. Evolution of a primary chicken myoblasts culture along a 48h period. Scale bar, 500 µm.

Supplementary Movie 4: Evolution of the myoblast culture transduced with the lentiviral construct allowing to observe actin (green) and myosin-II (magenta) cytoskeleton, associated to Hoechst staining (blue) to observe the nuclei, along a 48h period. Scale bar, 200 µm.

Supplementary Movie 5: Close-up area illustrating progressive myotube formation through fusion of myoblasts. Actin (green) and myosin-II (magenta) and nuclei (blue). Scale bar, 100 µm.

Supplementary Movie 6: Close-up area illustrating fusion events occurring during myotube formation. Actin (green) and myosin-II (magenta) and nuclei (blue). White arrowhead highlights actin foci preceding membrane merging and fluorescent signal mixing during the fusion process. Scale bar, 100 µm.

Supplementary Movie 7: Evolution of average isotropic stress maps (Pa. µm) obtained for +1/2 defects present at each analyzed time point in the 3 movies (48h) analyzed.

Supplementary Movie 8: Evolution of average isotropic stress maps (Pa. µm) obtained for -1/2 defects present at each analyzed time point in the 3 movies (48h) analyzed.

Supplementary Movie 9: Movie of single simulation realization showing the evolution of the active nematic system with coupling to ECM. Right: isotropic stress (color bar) and director (black lines) of nematic. Left: director of fibronectin (blue lines). White and black circles denote +1/2 and -1/2 defects respectively.
