# Supplementary material for: Publisher Correction: Extracellular-matrix mediated stresses spatially bias myoblast fusion and myotube growth
Source: Nat Commun. 2026 Oct 29;17:11251. doi: 10.1038/s41467-026-79196-z (PMC13720112; doi:10.1038/s41467-026-79196-z)
Supplement: Supplementary file 1 [file 41467_2026_79196_MOESM1_ESM.pdf]

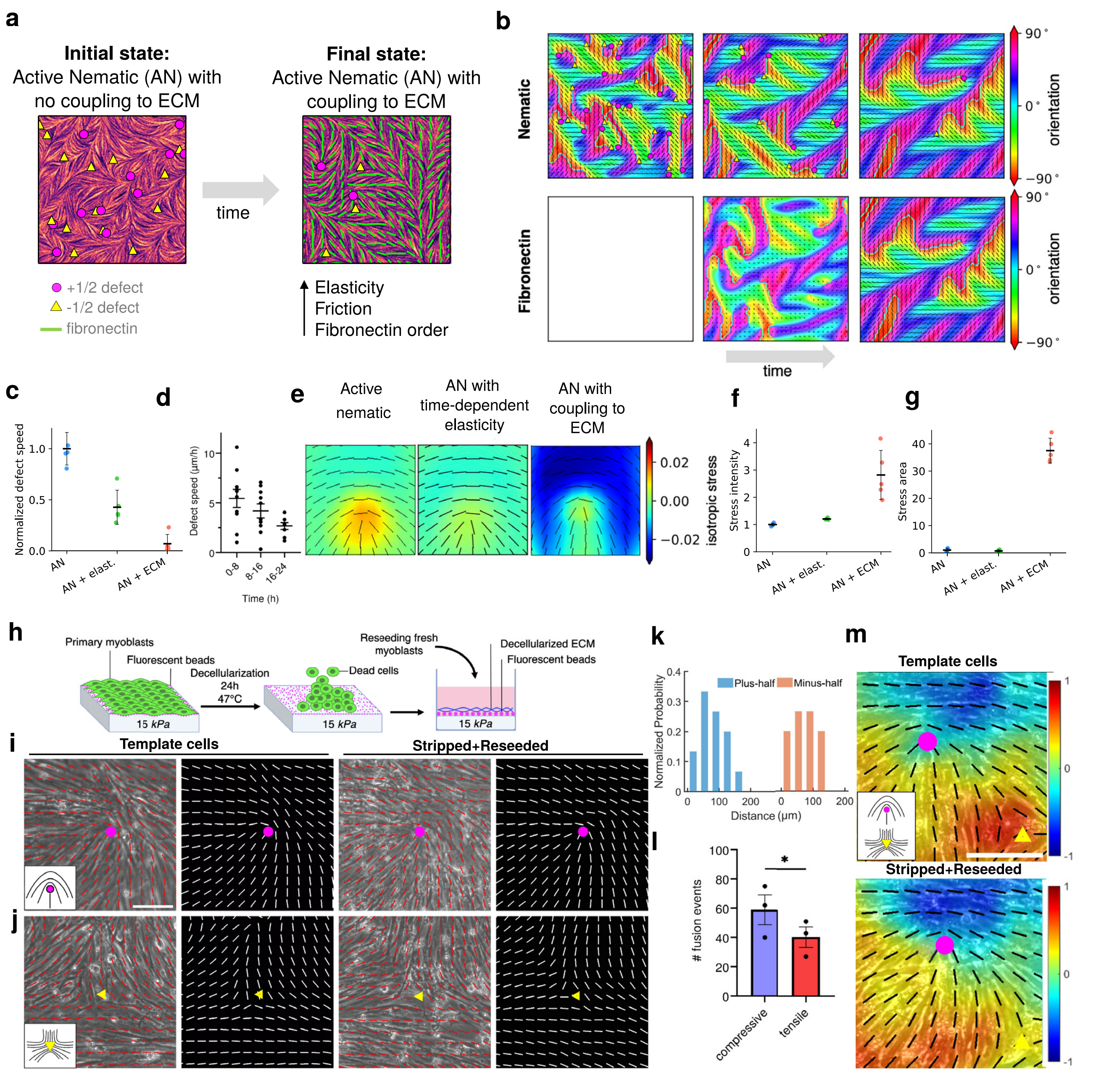

Fig. 4 | Change in the orientational elasticity and coupling to ECM dictates the stress patterns around defects. a Schematic representation of the modeling framework with time-dependent orientational elasticity and ECM evolution. Once system reaches active turbulence at t_0, the elasticity, friction and fibronectin order increase monotonically until saturation at maximal value. **b** Simulation snapshots of nematic (top) and fibronectin (bottom) orientation at different times of the simulation. **c** Defect speed for active nematic (AN) with no coupling to ECM, AN with time-dependent elasticity, AN with time-dependent elasticity coupling to ECM. The values are normalized by the mean defect speed in the AN case. Points correspond to independent simulations with different random seeds ($n = 5$); horizontal lines and error bars show the mean ± standard deviation. Relative to the AN case, the defect speed is significantly reduced for AN + elasticity ($^{***}p = 5.5 \times 10^{-4}$) and AN + ECM ($^{****}p = 1.9 \times 10^{-5}$) ($p$ values obtained via two-sided Welch's t test). **d** Defect speed from experiments at various time periods during the culture period ($n = 10$ stable defects, $^{*}p = 0.04$). **e** Average isotropic stress around +1/2 defect in active nematic (AN), AN with time-dependent elasticity and AN with time-dependent elasticity coupling to ECM. **f–g** Stress intensity (**f**) and area (**g**) at the head of the +1/2 defect in active nematic (AN), AN with time-dependent elasticity and AN with time-dependent elasticity coupling to ECM. The values are normalized by the mean values in the AN case. Both stress intensity and area are increased for AN + ECM ($^{**}p = 1.0 \times 10^{-2}$ and $^{****}p = 4.2 \times 10^{-5}$) ($p$ values obtained via two-sided Welch's t test). **h** Schematic of stripping/reseeding procedure: After nematic organization and traction fields were established, myoblasts were removed while preserving the underlying ECM, and fresh myoblasts were reseeded onto the stripped matrix (Created in BioRender. Dubey, S. (2026)). **i**, **j** Snapshots of cultures before stripping (Template) and after reseeding (Stripped+Reseeded) showing the reappearance of plus half (**i**) and minus half (**j**) nematic defects at the same locations as in the template culture. Alternating panels show the corresponding orientation vector fields. **k** Normalized probability density to observe the same topological defect after reseeding as a function of the position around +1/2 and −1/2 defects in the template culture. **l** Number of fusion events in compressive versus tensile regions after reseeding ($n = 298$ fusion events, averaged over 3 independent movies, significance was calculated by paired two tailed t-test $^{*}p = 0.0278$). **m** Isotropic stress map around defects in template (top) and stripped+reseeded matrix (bottom), overlaid with the averaged local orientation of the cells (black lines). **i**, **j**, **m** magenta dots and yellow triangles respectively mark the core of +1/2 and -1/2 defects and insets are schematics of the orientation field characteristic of +1/2 and −1/2 defects. **c**, **d**, **f**, **g**, **l** Error bars represent standard error (S.E.) from the mean. Source data are provided as a Source Data file.
